# AI Concepts for System of Systems Dynamic Interoperability

**DOI:** 10.3390/s24092921

**Published:** 2024-05-03

**Authors:** Jacob Nilsson, Saleha Javed, Kim Albertsson, Jerker Delsing, Marcus Liwicki, Fredrik Sandin

**Affiliations:** Embedded Intelligent Systems LAB (EISLAB), Lulea University of Technology, 97187 Lulea, Sweden; jacob@flasheye.se (J.N.); kim.albertsson@ltu.se (K.A.); marcus.liwicki@ltu.se (M.L.); fredrik.sandin@ltu.se (F.S.)

**Keywords:** system of systems, dynamic interoperability, AI for cyber-physical systems, representation learning

## Abstract

Interoperability is a central problem in digitization and System of Systems (SoS) engineering, which concerns the capacity of systems to exchange information and cooperate. The task to dynamically establish interoperability between heterogeneous cyber-physical systems (CPSs) at run-time is a challenging problem. Different aspects of the interoperability problem have been studied in fields such as SoS, neural translation, and agent-based systems, but there are no unifying solutions beyond domain-specific standardization efforts. The problem is complicated by the uncertain and variable relations between physical processes and human-centric symbols, which result from, e.g., latent physical degrees of freedom, maintenance, re-configurations, and software updates. Therefore, we surveyed the literature for concepts and methods needed to automatically establish SoSs with purposeful CPS communication, focusing on machine learning and connecting approaches that are not integrated in the present literature. Here, we summarize recent developments relevant to the dynamic interoperability problem, such as representation learning for ontology alignment and inference on heterogeneous linked data; neural networks for transcoding of text and code; concept learning-based reasoning; and emergent communication. We find that there has been a recent interest in deep learning approaches to establishing communication under different assumptions about the environment, language, and nature of the communicating entities. Furthermore, we present examples of architectures and discuss open problems associated with artificial intelligence (AI)-enabled solutions in relation to SoS interoperability requirements. Although these developments open new avenues for research, there are still no examples that bridge the concepts necessary to establish dynamic interoperability in complex SoSs, and realistic testbeds are needed.

## 1. Introduction

Systems within all industries are undergoing a rapid digitalization process associated with developments like industry 4.0 and the Internet of Things (IoT). Digitalization efforts are moving computing frameworks from monolithic computer systems to distributed computer systems consisting of thousands of computing elements, sensors, and actuators. The ambition is that production systems can become increasingly flexible and efficient thanks to increased connectivity, which increases the demands on the integration of software and physical components and the capacity to process data from a large variety of sources.

Several architectures have been developed to address the challenges of the digital industry, e.g., Reference Architectural Model Industrie 4.0 (RAMI 4.0) [1] and Industrial Internet Reference Architecture (IIRA) [2]. Implementations of such architectures is to a large extent based on service-oriented architecture frameworks like, e.g., Eclipse Arrowhead [3], FiWare [4], Eclipse Basyx [5], and LWM2M [6]. Using these frameworks it is possible to engineer SoSs consisting of many systems that can cooperate autonomously [7,8,9], provided that interoperability requirements are met. This way, monolithic computing architectures are gradually transformed into distributed computing systems, typically in the form of microservices [10]. The strategic relevance of SoS is described in roadmaps such as the 2022 Electronic Components and Systems (ECS) Strategic Research and Innovation Agenda (SRIA), but so far, there are few papers that present comprehensive examples of SoS engineering. See [11] for an example of a system of systems engineering (SoSE) methodology applying the Arrowhead framework in a smart city use case.

Moving from monolithic automation systems to distributed CPSs that can form SoSs introduces many challenges, of which dynamically establishing interoperability is the focus of this paper. Interoperability describes systems’ ability to use and exchange information [12], which is a prerequisite for the formation of a SoS. Large-scale SoSs are naturally expected to be heterogeneous, using different communication standards, semantic definitions, and state representations. Heterogeneity complicates an already complex problem; if systems use incompatible communication protocols and definitions, they cannot communicate and meet the requirements of a SoS without a translation or learning mechanism. Interoperability problems can occur at any point in the communication process, e.g., at protocol level [13].

### 1.1. Contribution

Here, we focus on dynamic interoperability in SoS including CPSs in the form of microservices [10] communicating with plaintext formats such as JSON and XML over arbritrary application-level protocols, with emphasis on learning-based approaches and related concepts AI, which also involves semantic interoperability of human-defined symbols within the constituent CPS and exchanged messages [14]. We also summarize developments of learning-based and emergent communication in the more general context of multi-agent systems, see [15] for an overview. Thus, our main contribution is a summary of recent advances in a previously disconnected body of literature addressing complementary aspects of the SoS (or agent) dynamic interoperability problem.

While interoperability between standards and protocols mostly has to do with the exchange of data (making sure that symbols in messages arrive correctly), interoperability at the message exchange level is also about *translating* the communicated information into symbol relations that the receiving system can make use of when performing tasks. This includes the problem of semantic translation, see, for example, [16,17], and extends beyond that since the system needs to update its state to maximize some form of utility or reward function given the new information about the state of the environment in the message payload data.

### 1.2. Study Scope

This review article is a combination of A narrative or traditional literature review and systematic literature reviews (SLR). Narrative reviews do not usually adhere to a strict protocol, and the selection of articles to include can be more subjective. SLR aims to provide evidence-based answers and often inform guidelines and policy. In contrast, narrative reviews aim to give a broader overview of a topic and are often more exploratory or introductory. We have utilized the best of both approaches in order to find the knowledge gaps in the addressed multi-disciplinary topics. Moreover, in each step of the review, from article searching to selection, from methods analysis to further investigation, we use subjective and intuitive synthesis of the contents. Hence, this article is not designed around specific research questions as we focus on a broader review of the listed domains. We describe knowledge gaps and present a survey of the literature that offers new opportunities to combine and make use of both data and metadata in a learning-based approach to message translation/communication in SoSs. Regarding the scientific scope of the selected studies, we limit the scope to investigating interoperability on plaintext data formats like JSON and XML. Investigating the potential uses of AI and machine learning on lower-level protocols such as HTTP, MQTT, or OPC-UA is an interesting problem left outside the scope of this paper.

### 1.3. Background

The traditional way to ensure interoperability is to manually engineer an “adapter” that translates messages of one type to another, or an “integrator” that integrates information from messages of multiple types. This approach works well when the system contains few components and is static on long timescales, but it does not scale to meet the requirements of a large and dynamic SoS [18]. Instead, semantic web technologies [19] and ontologies have been used to establish dynamic interoperability and SoS-level engineering, where the ontologies provide definitions and associations needed to transform data into useful formats, see for example [16,20,21]. Such methods can prove that the data transformation is correct within the context of the given ontology. At first, attempts were made to create large and universal ontologies [22], but recent research has favored the use of smaller, specialized ontologies [23,24]. A problem with using ontologies for data transformation is that they do not allow for graceful degradation, and the transformation will either work or not, depending on what data elements are present. Moreover, there are limited ontologies that are standardized by official conventions (such as W3C) and several manufacturers designing IoT systems which may or may not adhere to standardized ontologies [21].

Such problems can be addressed by either extending current ontologies or creating new ones, leading to more standards to consider when designing a system, which introduces interoperability problems between ontologies. Furthermore, legacy systems lacking ontology-based definitions will either need to be adapted to or have a custom adapter created, which drives the engineering efforts and cost when constructing large-scale SoS using semantic technologies. A more general approach to SoS interoperability is needed to efficiently deal with missing and inconsistent information.

### 1.4. Learning-Based Approach

Deep learning is successfully used to solve problems that are difficult to address with rule-based approaches, thanks to the statistical power of large datasets and increasingly efficient computing architectures [25]. A notable example of such problems are translation tasks, where Neural Machine Translation (NMT) has been an active research field in recent years [26]. The main difference between using deep learning-based methods and rule-based methods is that the former learn rules from data in a top-down manner according to some goal/objective, whereas rule-based approaches are usually designed bottom-up through deterministic algorithms, though efforts have been made to use rule-based approaches together with machine learning (ML)-methods, e.g., cognitive networks and zero-touch functionality [27]. However, the accuracy of a deep learning model is heavily dependent on the quality and quantity of available data, and some models can behave stochastically, unlike rule-based approaches where the failure modes are related to inconsistent or incorrect definitions. Recent works integrating machine-learning techniques for message interoperability in SoS [28] and multi-agent systems [29] demonstrate that there is a developing interest in such techniques, but further work is necessary to make machine learning a viable approach to interoperability.

## 2. Research Methodology

**Figure 1:**
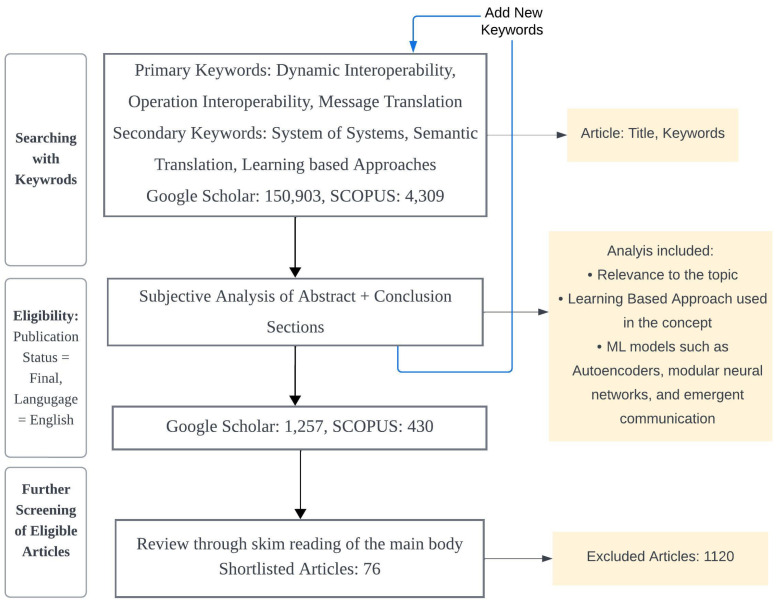
The flow diagram for the database search of publications for analysis.

This article is designed as a critical appraisal to look deeper into the indexed scientific literature in a variety of databases to highlight the existing knowledge gaps. The given topic is rather broad with a vast variety of applications across various domains due to which defining any specific summarized research questions was not a doable task to begin with. Therefore, we followed a qualitative research approach by first listing down the initial (Primary) keywords for article searching and then continued updating and adding new keywords (Secondary). We used Google Scholar and Scopus databases and a detailed flow chart of the process is shown in Figure 1. The synthesis of this article is purely based on subjective analysis in which the authors reviewed the articles and based on past experience and knowledge included or excluded the articles.

### 2.1. Structure of the Sections

**Figure 2:**
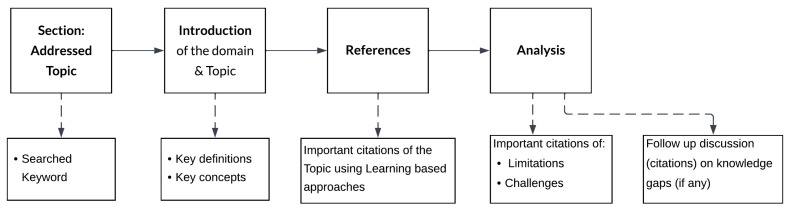
Layout of every section explicitly demonstrating the contents.

All the subjective knowledge presented in this article is drafted in a way to first elaborate on the background of every topic, then listing down the key concepts involved, next the technical details along with important citations, followed up by remarks on limitations and/or challenges of that topic. Lastly, we add a brief discussion on knowledge gaps if there are any. A breakout of sections is shown in Figure 2. Sections and their subsections that follow this layout are Section 4 and Section 5.

### 2.2. Search Results

Peer-reviewed articles were sourced from two data repositories, and literature searches were completed by 20 April 2023. These searches were conducted across various internationally recognized databases to gather pertinent information from publications. Scopus serves as a global database encompassing peer-reviewed publications worldwide. Google Scholar’s advanced search engine is beneficial for accessing citations that other databases do not include. Table 1 presents the search results.

## 3. Key Aspects of SoS Interoperability

The concept of SoSs was introduced to describe cooperation and collaboration among autonomously operating systems [7] whose operation is defined by five properties [8]:

1. Autonomy: constituent systems are functionally independent;
2. Belonging: constituent systems choose what SoS to belong to at run-time;
3. Connectivity: constituent systems can exchange information with each other at all times;
4. Heterogeneity: constituent systems use heterogeneous technologies and software interfaces;
5. Emergence: constituent systems cooperate to exhibit new or improved functionality related to SoS-level goals.

These five properties describe SoSs as constantly evolving systems that can assimilate new and previously unknown systems, find and use novel configurations to improve efficiency, or solve new tasks while not compromising the goals and functions of individual constituent systems. The complex interactions expected within a SoS create a complex environment for interoperability, potentially increasing time-to-deployment and costs due to current methods relying on hand-made adapters and standards [30]. Thus, to enable the large-scale SoS envisioned by, e.g., RAMI 4.0 and IIRA, automatic interoperability solutions must be developed.

The cost and complications of these aspects also apply to Systems of Cyber-Physical Systems (SoCPSs), SoSs consisting of CPSs, where Operational Technology (OT) and Information Technology (IT) should seamlessly integrate, and communication errors could propagate and cause physical systems to malfunction [18]. The cause of these errors is the *symbol grounding problem*, mismatches between symbolic models in the cyber domain, and the physical reality those models represent [31]. The symbol grounding problem is well known in robotics but is rarely highlighted in interoperability research. Symbol grounding could, in principle, be introduced by using an AI solution that can learn from the messages passed around in a SoCPS [32]. Such an AI solution, which can utilize and handle the semantic, dynamic, and operational requirements of a system, can achieve *automatic interoperability*, i.e., interoperability with minimal human input during operation. Automatic interoperability between constituent systems is a goal of SoSE and must comply with the properties of SoS stated above. To aid in the analysis of AI approaches to dynamic interoperability, we have developed seven requirements that an autonomous SoCPS should fulfill:

- Autonomy: constituent systems should be able to pursue system-level goals independently.
- Runtime Operation: new systems and policies should be integrated and responded to swiftly and appropriately.
- Fault Resilience: a SoCPS should gracefully adapt, potentially with degraded functionality, in the event of errors or changes in the constituent CPSs.
- Information Integration: all available data/metadata sources and knowledge representations should be utilized.
- Resource Efficiency: a SoCPS should implement the policies efficiently to minimize costs, including both monetary and environmental aspects.
- Security: privileged information should not be leaked to unprivileged parties.
- Generality: a proposed solution should be applicable to a wide variety of SoCPSs, thus being standard agnostic.

## 4. Interoperability Overview

Interoperability is the broad study of how systems can share and utilize shared information [12], and SoS interoperability is a broad subject that, in practice, includes aspects ranging from physical processes to security requirements. The main objective of this section is to describe the different roles and functions interoperability plays, not to introduce a new definition of interoperability. In this section, we present work done in the fields of *semantic*, *dynamic*, and *operational interoperability*, with a focus on machine-to-machine (M2M) communication and the related knowledge gaps [33].

### 4.1. Semantic Interoperability

Semantic interoperability is the interoperability of structured information artifacts, or semantic assets [34,35]. The components of semantic assets gain meaning from their structure and the context in which they are used, e.g., a list of two floating point numbers gain the meaning of coordinates when used in the context of geography. Similarly, a list of three strings could also mean two coordinates with an extra string defining the format of the first two strings. These two lists are semantically equivalent given a function that can relate these two lists as containing the same information.

Semantic assets can take many different forms, the aforementioned list is an example. Ontologies are a widely studied form of semantic asset, where the information forms a hierarchy of named/defined concepts and relations using first-order logic, providing the information required to perform inference on symbolic data, see for example [36]. From here on, when referring to semantic interoperability, we assume that the semantic asset is an ontology of some kind.

Ref. [36] addresses semantic interoperability by designing a service composition system that enables the goal-driven configuration of smart environments for end users. The new engineered system combines semantic metadata and reasoning with a visual modeling tool. The key feature they proposed is the embedded semantic services descriptions used for dynamically creating service mashups as per users’ goals.

Further, in another work the authors presented an Open Semantic Framework (OSF) to address the interoperability challenges of how to make sense of all the connected resources within a Web of Things (WoT) to create intelligent systems [23]. Their designed OSF is built on an extensible set of core ontologies that is designed to capture concepts from across domains. The core ontologies get integrated with domain-specific knowledge packs (KPs) that enable specific applications to access their required information. After testing their model on a use case, integrated with real-time data acquisition, the authors demonstrated that OSF could successfully provide straightforward access to knowledge models which could codify complex constraints for workplace safety and only exposes it through a moderated query API. Although these designs aim to achieve interoperability across the IOT Silos, when it comes to an extendable generically and automatically applicable solution for more than one use case, such solutions are still under systematic development.

**Figure 3:**
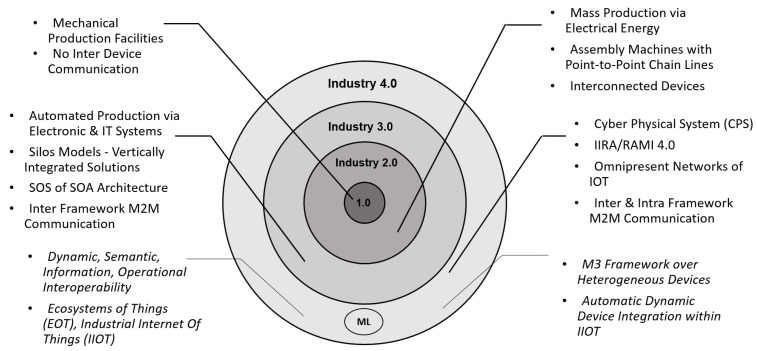
Key features of The Industrial Revolution from Industry 1.0 to Industry 4.0, including some features that ml adds to the recent development.

These days we find semantic interoperability state-of-the-art in several dimensions such as semantic annotations, compatibility verification, and generation of translators for XML messages across a heterogeneous framework [16]. Likewise, several solutions are built for Machine-to-Machine Communication (M2MC) based on annotation rules for a system’s meta-data that enables translation of data exchanged across heterogeneous devices in IoT [37]. A brief summary of existing models is presented in Figure 3 in which they are mapped onto the four tiers of industrial revolution. Clearly one can see that ML brought much more to the table in Industry 4.0.; however, whether any solution will work on more complex schema or not is still an open question. Currently, many models (discussed above) are limited to XML and lack the ability to incorporate JSON or multiple schemas.

Ref. [29] presents a solution for interoperability between a base context defined in the FIPA-ACL language and agent-specific context described with ontologies. The proposed architecture uses neural networks to decode messages into a list of components that are further analysed to decide agent states, for example “Permanent danger”.

### 4.2. Dynamic Interoperability

In statically configured systems, interoperability can be solved at design time and need not be considered until the system is modified. However, SoSs are dynamic because systems can change characteristics over time and may join or leave the SoS at any time. In particular, new, previously unknown systems should be able to join SoS and co-operate without requiring an engineer for manual system reconfiguration. This is known as *dynamic interoperability*, which is different from establishing and maintaining interoperability between static and slowly changing systems.

Dynamic interoperability is about “how” a system works, and not about what technology is used to enable interoperability. An example is the Internet Protocol, which enables interoperability on the internet level of the TCP/IP stack for diverse systems. A previously unknown system can connect to a router and now have access to billions of other devices on the internet. In SoS research, dynamic interoperability has been investigated in different ways, see e.g., [13] for dynamic protocol interoperability and [16] for dynamic semantic interoperability, both of which work by means of translating between protocols and ontologies.

### 4.3. Operational Interoperability

Operational interoperability is the ability of systems to cooperate and achieve goals which cannot be achieved by any individual system. Operational interoperability is important for coordination and orchestration of constituent systems to perform goals on a higher level than their autonomous goals. The term originally applied to human actors and their ability to cooperate [38], not that of software systems, but CPSs can be represented by both a human actor and an IT system. Examples of operational interoperability are cognitive networks [27], where the business intent is described using, e.g., an ontology; see [39], who designed a multi-agent systems architecture capable of detecting and mitigating anomalies in a manufacturing environment. The cognitive network will use the intent description and available data to decide what actions systems need to take, which is computed through a combination of reasoning and machine learning.

## 5. Towards Automatic Interoperability

**Figure 4:**
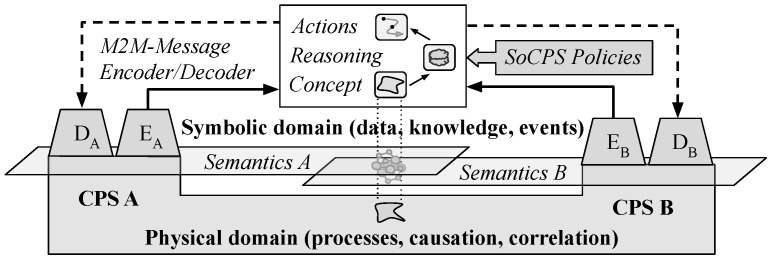
Example of SoCPS automation architecture in the case of two CPS. M2M message transcoding, concept learning, and policy-based reasoning are combined to automatically optimise the SoCPS and the concept representations (grounding) given a set of policies and the metadata/knowledge that defines the CPSs.

In this section we present work done in the AI/ML community that potentially can be adapted into new concepts and models enabling automatic interoperability. The development of machine learning methods for natural language processing, knowledge-based reasoning, and concept learning are timely opportunities to manage semantic heterogeneity in automation by aligning the physical and digital worlds by optimization, for example, as outlined in the architecture illustrated in Figure 4. In this architecture, M2M message transcoding, concept learning, and policy-based reasoning are combined to automatically optimise the SoCPS given a set of policies. The SoCPS is defined top-down by the policies, which optionally can include utility optimisation directives at the SoCPS level, as well as credentials and constraints. For example, energy production information available in CPS A could be part of a SoCPS energy-optimisation policy (collaborative benefit), or the predefined utility of CPS B can be optimised by information transfer from CPS A (cooperative benefit). The concept representations are grounded and semantically aligned to heterogeneous metadata definitions and knowledge (graphs) in each CPS, and the message/event encoders and decoders are optimized in a similar way as in natural language processing, see Section 5.2. The (neuro-symbolic) reasoning module require grounded concept representations to effectively deal with semantic heterogeneity, while maintaining an observable state throughout the optimisation process via semantic alignment to policies and knowledge defined in the CPSs. Such reasoning mechanisms are actively developed, for example in visual concept learning and question answering, see Section 5.5. While there are several open problems that need to be addressed before a functional architecture of this type that fully addresses the requirements specified in Section 3 can be realized, the rapid developments in the aforementioned machine learning areas create a timely opportunity to investigate and enable AI-based automation at the level of SoCPS. In the subsequent subsections, we outline the state-of-the-art approaches, provide an overview of each, and discuss their respective limitations.

### 5.1. Ontology Alignment

Ontology Alignment focuses on matching schemas of more than a single RDF dataset or Knowledge Graph (KG). Ontologies have become a vital approach to representing knowledge in a formal format. Over the years, many variants of ontology-based solutions have been developed. When it comes to dynamic interoperability among heterogeneous omnipresent networks built on multiple taxonomies, the challenge of semantically linking all ontologies arises [21,40]. Although, as per the Semantic Web Community guidelines, many ontologies are designed on similar syntax and semantics, they often differ considerably in various elements (e.g., naming or structure). Therefore, the automated process of discovering relations between representations of multiple ontologies has become relevant for designing applications in heterogeneous distributed environments. Theoretically, such alignment methods should have a number of necessary underlying attributes, a few of which are:

- Rational: representation of a real-world problem conceptualizing explicit specifications;
- Flexible: to be transferred to other applications;
- Extendable: can be utilized for other domains with different semantic structures;
- Dynamic: models should be applicable to run-time-operations of IoT frameworks;
- Automated Parameter Updates: must be boosted by ML techniques where weights or interpretation schemes are learnt and evolved;
- Visualization and Comparison: the model must be equipped with an illustrative representation of metadata and results where conclusive comparisons can be drawn.

There are several successful applications in the domain including [41] which addresses the problem of loosely connected pieces of information within Linked Open Data (LOD) where schema-level information gets ignored while building alignment-based solutions. Another model titled *BLOOMS* is based on instance-level ontology mapping and was designed around measuring the similarities between a pair of concepts according to the number of similar instance of ontology concepts [42]. Moreover, a significant work on sequence alignment-based ontology mapping architecture is presented in [43]. Their ontology mapping emphasises solving the problem of ontology heterogeneity by finding schema-level links in LOD for the Chinese Language. However, their solution is restricted to semi-automated applications as it requires a set of initial rules from which the model can learn further complex alignments. Likewise, so far every proposed solution specifically targets its domain and a continued challenge is to design a flexible model that can address all aspects of the problem.

### 5.2. Transcoder Architectures

**Figure 5:**
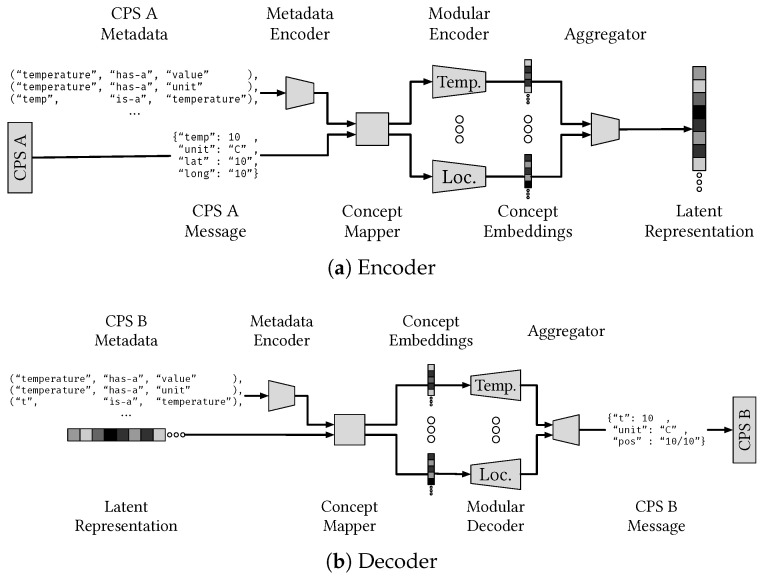
Example of a modular M2M transcoder architecture for structured messages, which integrates metadata for accurate and explainable transcoding of messages. The fields of the input messages, such as JSON, are mapped to concepts defined in an ontology by a concept mapper, and the resulting field–concept tuples are transcoded by specialized modules. The transcoded message fields are finally aggregated to complete messages that can be interpreted by the receiving CPS.

Translation of natural language and text often uses *transcoder* architectures, also called *encoder–decoder* architectures (e.g., [13,44,45]), see Figure 5.

In transcoders, the source text is fed to an encoder, which transforms the source to an intermediary format, and the translated text is reconstructed from the intermediary format using a decoder. For transcoders using neural networks to encode and decode, the intermediary format is a vector, often of a lower dimension than the input and output, which condenses or compresses the information in the source text. A special case transcoder is the *autoencoder*, which uses the same encoder–decoder architecture, but instead of translating data, the goal is to reconstruct the input [46]. Autoencoders are mainly used to generate embeddings—compressed representations of the input data—which can be used for data compression, feature extraction, or generation of new data [47]. Metadata embeddings are a particular kind of embedding, where the input to the autoencoder is, e.g., RDF-triplets [48] or Web Ontology Language (OWL) [49] data, which allows the use of metadata and knowledge graphs in neural networks.

Transcoder architectures have worked well for translation of natural languages where large parallel corpora, i.e., texts in multiple languages that share meaning, exist. However, parallel corpora cannot be obtained for all language pairs, which will likely also be true for the SoS translation task, and some other approach has to be used. Instead of training a transcoder on parallel data, *backtranslation* uses the shared structure of transcoders and autoencoders to train a translation model with few or no parallel examples. The idea is to first train autoencoders for one or more languages, then use backtranslation (translation from language A → B and then back B → A) to fine-tune the encoders and decoders for translation. Backtranslation shows promising results for translation on both natural languages and programming languages [50], while attempts to adapt backtranslation for message translation require further experimentation [28].

In the modular M2M message transcoder architecture illustrated in Figure 5, metadata are used to generate field–concept tuples that modular neural networks can encode into optimized concept embeddings. The decoder has a similar architecture but uses as input the latent representation and the metadata defining the symbols of, e.g., the receiving CPS. The latent representation is decomposed into concept embeddings, which are used by a joint decoder to reconstruct the message for the target system. The network modules can be defined automatically from the metadata and can be either specific to a particular system or taken from a pool of pretrained prototypes tuned through metalearning, allowing sharing of modules between systems. The role of modular neural networks, concept-based learning and reasoning, and metalearning are further described in the following subsections.

### 5.3. Modular Neural Networks

Modularity is an approach that provides flexibility to neural network design. Instead of training different models for different tasks end-to-end, modularity allows the network to be decomposed into task-dependent subnetworks. This is called *task decomposition*. Ref. [51] uses a modular approach to train policy networks for a small set of robotic arms performing a set of tasks. Each robotic arm and each task gets one subnetwork, which reduces the total amount of models needed to be trained compared to an end-to-end model (n+m vs. nm). In this model, the networks were composed, i.e., the input to the policy network was given to the task subnetwork, and the output of the task network was the input to the robotic arm subnetwork, whose output is the final output of the model.

Another approach to task decomposition presented in [52,53] is to have a single network that learns the number of subnetworks necessary, which adapts the architecture dynamically during training. This is done by having a task-decomposition layer that clusters the input and provides it to the task-specific subnetworks, whose output is combined in the next layer. The idea is that the task-decomposition layer will start with zero clusters and add more until some criterion is fulfilled, which is architecture specific.

Modularity and task decomposition are suitable approaches to neural network design for the dynamic interoperability task in SoSs. For the translation task, translating between a new pair of systems can be considered a new task, and if an encoder–decoder architecture is used, the encoder and decoder can be specialized for the systems in question. Further, the semantic metadata describing the messages, systems, and SoS in question could also be used to modularize a translator, as each metadata element describes how the system can interact with other systems, see Figure 5. If SoS-level goals are incorporated into the metadata, the modular approach could also help to achieve operational interoperability.

Choosing what modules are present in the network depending on the metadata used is a discrete view of modularity. Furthermore, as is often the case in neural network design, more granular approaches could be used. Consider the LSTM, a recurrent neural network that uses *gating* to choose what information gets preserved and what gets forgotten in the next time step [54]. A gating approach could be adapted, where the gating is dependent on the data, metadata, or both, and this approach would likely require the metadata to be embedded first, e.g., using OWL2Vec. One can also see the metadata as a data source for an *attention mechanism*, similar to techniques used in image captioning [55]. The idea is that each time step of an input sequence gets a summary of all previous inputs and an image and learns what part of the input and image the network should pay attention to [56]. In the interoperability case, the sequence would be a message, and the image is swapped with metadata elements.

### 5.4. Emergent Communication

The communication between learning agents in a system does not need to be established at design time. Instead, the communication protocols used can emerge from the behaviours of the learning agents and systems, a process called *emergent communication* in the field of multi-agent systems ([15], Section 5.2). Ref. [57] present early investigations in this field, using evolutionary algorithms to evolve agents that can combine a static set of sounds into words and sentences to describe objects.

Emergent communication has recently been getting more attention in the deep learning community. Ref. [58] present a metalearning approach to quickly converge the language of populations of pre-trained agents. The pre-trained agents serve as a prior for language evolution, and it is shown that both pre-trained networks and human language can be used as a basis for the metalearner.

Ref. [59] explores how agents can learn compositional languages, i.e., languages where the number of combinations of tokens and grammar rules is larger than the amount of tokens and grammar rules. They set up a speaker–listener game, where agents communicate via embeddings created by variational autoencoders [60] using discrete latent variables, and explore how model capacity and channel bandwidth affects the emergent language. They find that while there is a lower limit where bandwidth and capacity to where the emergent language is composite, an upper bound could not be determined.

Ref. [61] explores the effect of connectivities and group size on emergent language, using a model similar to [59]. They set up an experiment with two groups of agents using variational autoencoders, where the first group learns the shapes of objects, and the second group learns the color of objects. Agents from these groups can communicate, using an emergent language, and cooperatively perform the combined task of shape and color recognition. They find that by manipulating the size and shape of the communication graphs they are able to influence the formation of local varieties of emergent language, i.e., dialect formation. Despite the formation of dialects, agents of distant dialects can still effectively perform the combined task.

Ref. [62] investigates how the construction of discrete communication tokens affect communication learning. They provide theoretical arguments to show that using a static set of discrete communication tokens is not powerful enough to evolve emergent communication in general. Instead, they propose to use neural networks to learn an underlying semantic space, from which discrete communication tokens can be extracted. This technique generalizes better, and they show that the tokens learned are semantically meaningful to humans agents.

### 5.5. Concept-Based Learning and Reasoning

People can learn new concepts from just one or a handful of examples, whereas standard algorithms in machine learning typically require orders of magnitude more. In particular, people learn feature-rich representations of entities and their relations, constructed by composing simpler primitives, leading to a high capacity for generalisation [63,64]. Generalizing to novel tasks given only a few labeled examples is a fundamental challenge in closing the gap between machine- and human-level performance. A recent trend towards these goals is the explicit representation and manipulation of concepts in a paradigm known as Concept Learning. Approaches like these, where symbolic information is mapped to semi-structured grounded representations of entities and events in the world via an optimisation process is a natural way to bridge the gaps between semantics, data, and causal powers.

For example, Ref. [65] synthesises a computational graph where extracted object and concept representations are used as inputs. The nodes of the computational graph are taken from a pool of parameterless functions representing reasoning constructs such as functions for selecting an object, or relating the relative position of two objects. Ref. [66] further extends this definition to include learnable functions, metaconcepts, representing relationships between concepts: $f(C_{a},C_{b},R;\Theta )$, a learnable definition of the RDF triple.

In [64] a semantic reasoning structure is layered on top of a prototype-based network for image classification where the semantic reasoner provides an attention-like mechanism controlled by metadata. The model input is routed to specialised parameterised modules whose output is subsequently weighted according to a local importance score and aggregated. Extraction of concepts is done independently, there is no joint learning of concepts and their representation, and this can come from, e.g., ontological data. In this way the network can choose to include or exclude an entire concept at the abstract level, make use of fine-grained information in the concept representation, and explain the importance of each input concept for the final output.

Motivated by the restricted local receptive field of kernels in a convolutional network and the ability of humans to manipulate abstract semantic concepts, ref. [67] introduces a computationally efficient reasoning module with sparse interactions between concepts in a multi-branch architecture where each branch is taken to represent a concept. A key feature is the ability of the network to modulate the output of each branch depending on a non-local context vector computed by using an attention-based sampling mechanism to derive a succinct concept representation from the feature maps in each branch, which is further refined by a fully-connected graph operation. The resulting model is able to achieve competitive performance on a multitude of datasets with similar computational requirements as the backbone network. The concept extraction and modulation process is automatic and fully trainable (in contrast to [64]) but the connection to human-level concepts is less clear. The authors show that some feature maps seem to correspond to clear features, but not all concepts learned are easily interpretable; a direction not explored in the work is how these concepts can be connected to ontologies or knowledge bases.

An early example of concept-based learning and reasoning for SoSs automation is presented in [68], where concepts are represented in a vector-symbolic architecture and actions are automated using a combination of causality-based imitation learning and analogy making.

A related, but different, approach to symbol manipulation is provided by *logic reasoners*: programs executing a logical query on a knowledge base or ontology. These systems allow automatic answering of complex queries, yet it is unclear how to integrate them into a deep learning framework. A work in this direction is [69], where an approximate SAT solving layer is developed that discovers logical rules during the learning process; it can jointly learn input–output mapping and rules for that mapping based on the relationships between input variables and is shown to outperform a method based on local connectivity between input features.

Incorporating concept-based reasoning can produce models with better generalisation from less data, increasing resource efficiency and allowing translation to novel tasks facilitating runtime operation, both in part due to the increased level of abstraction. The model can benefit both from high-level relational information and fine-grained details in specific features. This is desirable for integration into SoSs, where gathering significant amounts of data for all possible devices is prohibitively expensive. In fact, all possible devices are not even defined at training time. Generalisation to novel tasks is a fundamental requirement for such systems. Additionally, explicit representation of concepts allows these to be manipulated directly, which could increase fault resilience and system robustness, for example, by mitigating internal representation drift in the online setting. There is opportunity to integrate the ontological metadata available for SoSs, an area currently under-explored. Ontologies, rich representations of data, could be used to inform model initialisation or extend the application of concept learning to simpler models. They furthermore allow model output to be expressed in terms of human-relatable concepts facilitating interpretability. Finally, incorporating differentiable logic reasoners, particularly when applied to the concept level where the input space is sparse, can provide top-down feedback that can be difficult or impossible to discern from a bottom-up approach.

### 5.6. Metalearning

In metalearning the target is to learn an algorithm capable of instantiating well-performing models given a task definition, metadata, and possibly by inspecting input data samples. This way, metalearning operates on a distribution of tasks in a systematic, data-driven way, leveraging prior experience to bootstrap performance in novel, but related, settings. AutoML and *k*-shot learning are two examples where metalearning is used to address problems that can be useful in addressing the M2M message transcoding problem described here.

AutoML was introduced to lower the threshold for applying machine learning to a given problem and aims to automate the selection and execution of an entire data processing pipeline comprising a large, structured parameter search space containing both differentiable and non-differenetiable parameters. Bayesian optimisation is an attractive option to effectively explore such a search space, however, without leveraging prior knowledge it is time-consuming. The search process can be warm-started by seeding it with pipelines known to perform well on similar tasks. Motivated by this, nearest-neighbour search in metafeature space is used [70], i.e., using summary statistics calculated on the dataset to select suitable pre-generated pipelines optimised to perform well in its given task. Calculating useful metafeatures can be time consuming, and some measures are ill-defined in the presence of, e.g., categorical data, reducing their applicability. As an alternative to warm-start Bayesian optimisation, when a full search is prohibitively costly to run, recent efforts, [71], adopt *portfolios*, pre-computed, complementary pipelines that perform robustly across a large set of tasks.

Training in machine learning is often data-intensive; humans, however, can effectively learn new tasks without a even a single training example, relying on a task description and prior knowledge. This is the main motivation behind *k*-shot learning in which the model is only allowed to sample *k* examples from the input data. A general approach is presented in [72,73] which has been successfully applied in regression, classification, and reinforcement learning settings. The objective is to find a location in parameter space which is easy to specialise, where the latter uses a more expensive second-order approach with comparable performance. The initial model parameters Φ are updated using a fixed number of gradient descent iterations yielding parameters *W*. The initial parameters Φ are then updated with a small step in the direction of *W*: Φ←(1−ϵ)Φ+ϵW. In this way the final model parameters Φ are well-suited for rapid adaptation with only a few steps of gradient descent.

A special case, zero-shot learning, has k=0 forcing the metamodel to rely only on prior knowledge provided by auxiliary data sources such as knowledge bases or ontologies. In [74], semantic vector representations of class labels are refined by incorporating a relational network and predefined knowledge bases to address problems of representations, e.g., polysemic words and handling multi-word label.

An extension of the *k*-shot learning problem is the generalised setting [75,76], where the definition is changed to include both novel and seen classes during evaluation to better match real-world applications. Existing techniques in both zero- and few-shot learning fail to establish a globally consistent label space; when presented only unseen classes discrimination is successful, but when the joint label space is used models show a clear bias towards seen classes.

AutoML explores the setting where large amounts of data are available, while *k*-shot learning focuses on limited-data tasks. Zero-shot learning must, by construction, rely on metadata and auxiliary data sources. For example, the integration of ontological data is actively explored. In heterogeneous SoCPS we cannot expect to have access to training data from all systems and will see a mixture of both previously known and unknown systems. Thus, techniques pertaining to the generalised *k*-shot problem setting are relevant.

## 6. Open Questions and Future Research Challenges

### 6.1. Development of ML-Based Methods for SoS Interoperability

The task to dynamically establish interoperability between SoCPS in heterogeneous environments is a challenging problem. Different standards, semantic models, and legacy systems result in an inconsistent mix of semantic spaces, which are non-static in the presence of, e.g., reconfigurations, software updates, technology migration, and maintenance. Thus, the mainstream approaches to establish interoperability via standardization and translation at the symbolic/metadata level using, e.g., JSON and XML plaintext messages, have limited automation and scalability potential, which motivates further research on ML-based methods in this context. This observation motivates the survey of ML methods in Section 5, which can be helpful to address the general SoCPS dynamic interoperability problem. The concepts and methods described, like metadata-driven concept learning and metalearning, represent prominent examples that have proven useful to address real-world tasks that share some qualities with the SoCPS dynamic interoperability problem. In particular, transcoder architectures, like that illustrated in Figure 5, have proven effective for translation problems in several domains [44] and require only one encoder–decoder pair for each system, O(n), as opposed to each system pair, $O(n^{2})$, thus requiring less resources and training data. Additionally, such an architecture allows for integrating system metadata, for example, by letting each encoder–decoder pair receive metadata embeddings as input. Taking a modular ML model approach would allow for decomposition of the problem into smaller parts.

However, several details of a fully functional architecture of the type outlined here remain to be investigated, and the set of ML-based automation opportunities surveyed here are limited to some particularly evident cases and should not be considered a comprehensive review, especially considering the rapid progression of the research on these topics. While the above aspects are being examined in isolation in an increasing body of research, the application to dynamic interoperability in the SoCPS setting requires their joint intersection, which is an area essentially missing in the literature with only a few recent contributions, see for example [28] and references therein. The architecture for M2M communication outlined in Figure 5 and related discussion of the SoCPS interoperability problem define some general research directions, but the details of the implementation remains to be understood and developed. For example, the data and metadata are often textual representations of a graph structure, which means that both character-, word-, and graph-level models could be used to encode such data, but there is yet no consensus on the best approach. Furthermore, the aggregating and concept mapping steps in our proposed model (Figure 5) could also be done in different ways, either as fully trainable ML models, logical predefined models, or a mix of predefined and trainable components. The modular encoder part has similar open problems; what concepts and tasks should have their own modules, and should the modules always be the same or should they depend on the translation target? While the modularization approach seems reasonable in principle, there are open questions regarding architecture and efficient optimization protocols.

A solution of the SoCPS dynamic interoperability problem based on policies and heterogeneous system utility definitions will likely require a (federated) reinforcement learning protocol for the reasoning process outlined in Figure 4, possibly in combination with digital twins needed for safe parameter tuning and validation. This is further complicated by the numerous standards, data/metadata formats, and LOD datasets with loosely connected pieces of symbolic information which need to be dynamically integrated in a SoCPS. Recent work on emergent and learning-based communication in the context of agent based-systems can provide some guidance, such as the agents that learn to communicate in a simple game using symbols that are also meaningful for humans [62]. Further work is necessary to map the basic research on emergent communication in agent-based systems to the SoS interoperability problem, particularly in realistic scenarios involving complex heterogeneous definitions of the individual systems.

### 6.2. SoCPS Engineering Challenges

While the challenges of creating ML models suitable for SoCPS interoperability are themselves great, there will also be practical challenges involved with deploying ML in SoCPS. Here we highlight the prominent ones:

1. **Lack of open datasets:** There is a lack of open datasets suitable for end-to-end optimization and potential logistical and practical issues with gathering data from business-critical automation systems. Addressing these engineering issues requires further work, which could involve sharing digital twins and developing open testbeds.
2. **Privacy and security concerns:** Privacy and security aspects are critical for the transfer of new SoCPS technologies developed in such model environments to real-world applications, and the problems to establish privacy and security while allowing for systems to share the information required for learning and optimization are challenging. These challenges are partially addressed in the fields of federated ML and ML under fully homomorphic encryption, see for example [77,78], but the solutions developed need to be adapted to the SoCPS domain.
3. **Lack of protocols for production environments:** Furthermore, the training and validation protocols developed need to be aligned with the requirements of production environments, and a working culture which often emphasises the importance of explainable and predictable solutions. This includes developing effective exception handling solutions for graceful degradation in the case of M2M communication failures, etc.

## 7. Conclusions

In this paper, we describe the problems within establishing dynamic interoperability in SoCPS and present a literature survey of AI and ML approaches that could potentially be used in automatic interoperability solutions.

The results of the survey are summarized in Table 2, where we have organized the papers by the kind of interoperability their approach establishes (dynamic/static) or if the article discusses general AI concepts useful for SoCPS interoperability. We further note if the approach uses a rule-based (symbolic) or learning-based (subsymbolic) approach, if the approach utilizes goal-based cooperation, and if they consider fault resiliency and resource efficiency. Subsymbolic approaches have increased in popularity recently and we have identified nine papers that show interesting research and approaches, namely [23,28,29,43,58,59,61,62,68]. Not all of these papers mention interoperability explicitly, due to research groups working in separate fields with different goals in mind. Nevertheless, we think that the problems these papers are investigating are fundamentally linked to the interoperability, and combining these approaches is essential to address the dynamic SoCPS interoperability problem.

While there are several open problems that need to be addressed before a generic architecture of the kind outlined in Figure 4 and Figure 5 can be realised, see Section 6, the rapid developments in the aforementioned machine-learning areas creates a timely opportunity to investigate and develop AI-assisted dynamic interoperability solutions for scalable SoCPS engineering. Such technology is key given the present development of operational technology and production value chains, which require a shift towards large-scale optimization and SoS solutions, as well as for dealing with societal challenges that require large-scale system coordination and optimisation. This development drives the invention of many new standards, data/metadata formats and symbolic definitions, which increases system heterogeneity and scalable SoCPS technologies thus need to become increasingly standard invariant. In general, since the number of communicating systems will far outnumber people on the planet, and our society depends on their cooperation, a take-away message for machine learning researchers is that machine language processing in heterogeneous environments deserves more attention. Recent progress on other challenging tasks, like visual concept learning and multimodal transcoding using for example combinations of encoder–decoder networks and generative adversarial networks (GANs), are encouraging for addressing the SoCPS dynamic interoperability problem with similar ML concepts. This requires development of appropriate open data sets, simulators (digital twins) and test beds to support the advancement of models and solutions.

## Figures and Tables

**Table 1:** Some of the search terms used and the total number of publications from each database.

Databases	Searching Strings and Keywords		Number of Articles		Date of Acquisition
			*Title, Abstract, Keywords*	*Entire Article*	
**Scopus**	Primary:	Dynamic Interoperability, SoS, Learning based Approach	1876	3520	20 April 2023
	Secondary:	Operation Interoperability, Semantic Translation, Cyber Physical Systems	2556	4309	20 April 2023
		Emergent Communication, Ontology Alignment, Message Translation	3965	4277	20 April 2023
**Google** **Scholar**	Primary:	Dynamic Interoperability, SoS, Learning based Approach	50,978	150,903	20 April 2023
	Secondary:	Operation Interoperability, Semantic Translation, Cyber Physical Systems	45,881	90,034	20 April 2023
		Dynamic Interoperability, SoS, Learning based Approach	61,124	54,901	20 April 2023

**Table 2:** Summary of system/agent interoperability aspects and AI concepts considered in the reviewed literature (sorted in columns by order of succession).

References	Run-Time (Dynamic) vs. Pre-Defined (Static) Interoperability, or Generic AI Concept	Rule-Based (Symbolic) or Learning-Based (Subsymbolic)	Utility- or Goal-Based Cooperation (Yes/No)	Fault Resilience Aspects (Yes/No)	Resource Efficiency Aspects (Yes/No)
[13]	static	symbolic	no	no	yes
[44]	static	symbolic	no	yes	yes
[42]	static	symbolic	yes	no	yes
[41]	static	symbolic	yes	no	yes
[16]	static	symbolic	yes	yes	yes
[29]	static	subsymbolic	no	no	no
[69]	dynamic	symbolic	no	no	no
[70]	dynamic	symbolic	no	no	no
[71]	dynamic	symbolic	no	no	no
[72]	dynamic	symbolic	no	no	yes
[73]	dynamic	symbolic	no	no	yes
[36]	dynamic	symbolic	yes	no	no
[16]	dynamic	symbolic	yes	no	no
[57]	dynamic	symbolic	yes	no	no
[39]	dynamic	symbolic	yes	yes	no
[27]	dynamic	symbolic	yes	yes	yes
[28]	dynamic	subsymbolic	no	no	yes
[68]	dynamic	subsymbolic	no	yes	no
[29]	dynamic	subsymbolic	yes	no	no
[58]	dynamic	subsymbolic	yes	no	no
[23]	dynamic	subsymbolic	yes	no	yes
[43]	dynamic	subsymbolic	yes	no	yes
[61]	dynamic	subsymbolic	yes	yes	no
[62]	dynamic	subsymbolic	yes	yes	no
[59]	dynamic	subsymbolic	yes	yes	yes
[48]	concept	subsymbolic	no	no	no
[45]	concept	subsymbolic	no	no	no
[49]	concept	subsymbolic	no	no	no
[67]	concept	subsymbolic	no	no	no
[65]	concept	subsymbolic	no	no	yes
[66]	concept	subsymbolic	no	no	yes
[63]	concept	subsymbolic	no	no	yes
[76]	concept	subsymbolic	no	no	yes
[64]	concept	subsymbolic	no	no	yes
[74]	concept	subsymbolic	no	yes	no
[52]	concept	subsymbolic	yes	yes	yes
[51]	concept	subsymbolic	yes	yes	yes
[53]	concept	subsymbolic	yes	yes	yes

## References

[1] Hankel M., Rexroth B. (2015). The Reference architectural model industrie 4.0 (RAMI 4.0). ZVEI.

[2] Lin S.W., Miller B., Durand J., Bleakley G., Chigani A., Martin R., Murphy B., Crawford M. (2017). The Industrial Internet of Things Volume G1: Reference Architecture.

[3] The Eclipse-Arrowhead Consortium (2020). Eclipse-Arrowhead. Arrowhead Official Website. www.arrowhead.eu.

[4] Fiware (2020). FIWARE. Fiware: The Open Source Platform for Our Smart Digital Future. www.fiware.org.

[5] BaSys (2020). Eclipse BaSyx. www.eclipse.org/basyx.

[6] OMA (2020). OMA SpecWorks. Lightweight M2M (LWM2M). https://omaspecworks.org/what-is-oma-specworks/iot/lightweight-m2m-lwm2m/.

[7] Maier M.W. (1998). Architecting principles for systems-of-systems. Syst. Eng. J. Int. Counc. Syst. Eng..

[8] Boardman J., Sauser B. System of Systems-the meaning of of. Proceedings of the 2006 IEEE/SMC International Conference on System of Systems Engineering.

[9] Fortino G., Savaglio C., Spezzano G., Zhou M. (2021). Internet of Things as System of Systems: A Review of Methodologies, Frameworks, Platforms, and Tools. IEEE Trans. Syst. Man Cybern. Syst..

[10] Dragoni N., Giallorenzo S., Lluch-Lafuente A., Mazzara M., Montesi F., Mustafin R., Safina L. (2017). Microservices: Yesterday, Today, and Tomorrow. Present and Ulterior Software Engineering.

[11] Delsing J. (2021). Smart City Solution Engineering. Smart Cities.

[12] Geraci A., Katki F., McMonegal L., Meyer B., Lane J., Wilson P., Radatz J., Yee M., Porteous H., Springsteel F. (1991). IEEE Standard Computer Dictionary.

[13] Derhamy H., Eliasson J., Delsing J. (2017). IoT Interoperability—On-Demand and Low Latency Transparent Multiprotocol Translator. IEEE Internet Things J..

[14] Javed S. (2022). Towards Digitization and Machine learning Automation for Cyber-Physical System of Systems. Ph.D. Thesis.

[15] Gronauer S., Diepold K. (2021). Multi-agent deep reinforcement learning: A survey. Artif. Intell. Rev..

[16] Moutinho F., Paiva L., Köpke J., Maló P. (2018). Extended Semantic Annotations for Generating Translators in the Arrowhead Framework. IEEE Trans. Ind. Inform..

[17] Novo O., Francesco M.D. (2020). Semantic Interoperability in the IoT: Extending the Web of Things Architecture. ACM Trans. Internet Things.

[18] Nilsson J., Sandin F. Semantic Interoperability in Industry 4.0: Survey of Recent Developments and Outlook. Proceedings of the 2018 IEEE 15th International Conference on Industrial Informatics (INDIN).

[19] Shadbolt N., Berners-Lee T., Hall W. (2006). The semantic web revisited. IEEE Intell. Syst..

[20] Santipantakis G.M., Vouros G.A., Doulkeridis C., Vlachou A., Andrienko G., Andrienko N., Fuchs G., Garcia J.M.C., Martinez M.G. Specification of semantic trajectories supporting data transformations for analytics: The datAcron ontology. Proceedings of the 13th International Conference on Semantic Systems.

[21] Javed S., Usman M., Sandin F., Liwicki M., Mokayed H. (2023). Deep Ontology Alignment Using a Natural Language Processing Approach for Automatic M2M Translation in IIoT. Sensors.

[22] Smith B. (2012). Ontology. The Furniture of the World.

[23] Mayer S., Hodges J., Yu D., Kritzler M., Michahelles F. (2017). An open semantic framework for the industrial internet of things. IEEE Intell. Syst..

[24] Horsch M.T., Chiacchiera S., Seaton M.A., Todorov I.T., Šindelka K., Lísal M., Andreon B., Kaiser E.B., Mogni G., Goldbeck G. (2020). Ontologies for the Virtual Materials Marketplace. KI-Künstliche Intell..

[25] Halevy A., Norvig P., Pereira F. (2009). The unreasonable effectiveness of data. IEEE Intell. Syst..

[26] Ranathunga S., Lee E.S.A., Prifti Skenduli M., Shekhar R., Alam M., Kaur R. (2023). Neural machine translation for low-resource languages: A survey. ACM Comput. Surv..

[27] Niemöller J., Mokrushin L., Kumar Mohalik S., Vlachou-Konchylaki M., Sarmonikas G. (2020). Cognitive Processes for Adaptive Intent-Based Networking.

[28] Nilsson J., Delsing J., Sandin F. Autoencoder Alignment Approach to Run-Time Interoperability for System of Systems Engineering. Proceedings of the 2020 IEEE 24th International Conference on Intelligent Engineering Systems (INES).

[29] Amrani N.E.A., Youssfi M., Bouattane O., Abra O.E.K. Interoperability between Heterogeneous Multi-agent Systems Recommended by FIPA: Towards a Weakly Coupled Approach Based on a Network of Recurrent Neurons of the LSTM type. Proceedings of the 2020 3rd International Conference on Advanced Communication Technologies and Networking (CommNet).

[30] Delsing J. (2017). IoT Automation: Arrowhead Framework.

[31] Cubek R., Ertel W., Palm G. A Critical Review on the Symbol Grounding Problem as an Issue of Autonomous Agents. Proceedings of the KI 2015: Advances in Artificial Intelligence.

[32] Nilsson J., Sandin F., Delsing J. Interoperability and machine-to-machine translation model with mappings to machine learning tasks. Proceedings of the 2019 IEEE 17th International Conference on Industrial Informatics (INDIN).

[33] Gürdür D., Asplund F. (2018). A systematic review to merge discourses: Interoperability, integration and cyber-physical systems. J. Ind. Inf. Integr..

[34] Horsch M.T., Chiacchiera S., Bami Y., Schmitz G.J., Mogni G., Goldbeck G., Ghedini E. (2020). Reliable and interoperable computational molecular engineering: 2. Semantic interoperability based on the European Materials and Modelling Ontology. arXiv.

[35] Stevens R., Rector A., Hull D. (2010). What is an ontology?. Ontogenesis.

[36] Mayer S., Verborgh R., Kovatsch M., Mattern F. (2016). Smart configuration of smart environments. IEEE Trans. Autom. Sci. Eng..

[37] Campos-Rebelo R., Moutinho F., Paiva L., Maló P. Annotation Rules for XML Schemas with Grouped Semantic Annotations. Proceedings of the IECON 2019—45th Annual Conference of the IEEE Industrial Electronics Society.

[38] Sándor M. (2002). An analysis of basic interoperability related terms, system of interoperability types. Acad. Appl. Res. Mil. Sci..

[39] Mantravadi S., Chen L., Møller C. Multi-agent manufacturing execution system (MES): Concept, architecture & ML algorithm for a smart factory case. Proceedings of the 21st International Conference on Enterprise Information Systems, ICEIS 2019.

[40] Ehrig M. (2006). Ontology Alignment: Bridging the Semantic Gap.

[41] Jain P., Hitzler P., Sheth A.P., Verma K., Yeh P.Z. (2010). Ontology alignment for linked open data. Proceedings of the International Semantic Web Conference.

[42] Isaac A., Van Der Meij L., Schlobach S., Wang S. (2007). An empirical study of instance-based ontology matching. The Semantic Web.

[43] Wang T. (2019). Aligning the large-scale ontologies on schema-level for weaving Chinese linked open data. Cluster Comput..

[44] Maló P.M.N. (2013). Hub-and-Spoke Interoperability: An Out of the Skies Approach for Large-Scale Data Interoperability. Ph.D. Thesis.

[45] Vaswani A., Shazeer N., Parmar N., Uszkoreit J., Jones L., Gomez A.N., Kaiser Ł., Polosukhin I. (2017). Attention is all you need. Advances in Neural Information Processing Systems.

[46] Vincent P., Larochelle H., Lajoie I., Bengio Y., Manzagol P.A. (2010). Stacked denoising autoencoders: Learning useful representations in a deep network with a local denoising criterion. J. Mach. Learn. Res..

[47] Kusner M.J., Paige B., Hernández-Lobato J.M. Grammar variational autoencoder. Proceedings of the 34th International Conference on Machine Learning-Volume 70. JMLR. org.

[48] Ristoski P., Paulheim H. (2016). RDF2Vec: RDF graph embeddings for data mining. Proceedings of the International Semantic Web Conference.

[49] Holter O.M., Myklebust E.B., Chen J., Jimenez-Ruiz E. (2019). Embedding OWL ontologies with OWL2Vec. Proceedings of the CEUR Workshop Proceedings.

[50] Roziere B., Lachaux M.A., Chanussot L., Lample G. (2020). Unsupervised translation of programming languages. Adv. Neural Inf. Process. Syst..

[51] Devin C., Gupta A., Darrell T., Abbeel P., Levine S. Learning modular neural network policies for multi-task and multi-robot transfer. Proceedings of the 2017 IEEE International Conference on Robotics and Automation (ICRA).

[52] Qiao J., Zhang Z., Bo Y. (2014). An online self-adaptive modular neural network for time-varying systems. Neurocomputing.

[53] Li W., Li M., Qiao J., Guo X. (2020). A feature clustering-based adaptive modular neural network for nonlinear system modeling. ISA Trans..

[54] Hochreiter S., Schmidhuber J. (1997). Long short-term memory. Neural Comput..

[55] Xu K., Ba J., Kiros R., Cho K., Courville A., Salakhudinov R., Zemel R., Bengio Y. Show, attend and tell: Neural image caption generation with visual attention. Proceedings of the International Conference on Machine Learning.

[56] Anderson P., He X., Buehler C., Teney D., Johnson M., Gould S., Zhang L. Bottom-up and top-down attention for image captioning and visual question answering. Proceedings of the IEEE Conference on Computer Vision and Pattern Recognition.

[57] Nowak M.A., Krakauer D.C. (1999). The evolution of language. Proc. Natl. Acad. Sci. USA.

[58] Gupta A., Lowe R., Foerster J., Kiela D., Pineau J. Seeded self-play for language learning. Proceedings of the Beyond Vision and LANguage: inTEgrating Real-world kNowledge (LANTERN).

[59] Resnick C., Gupta A., Foerster J., Dai A.M., Cho K. (2019). Capacity, bandwidth, and compositionality in emergent language learning. arXiv.

[60] Kingma D.P., Welling M. (2013). Auto-encoding variational bayes. arXiv.

[61] Kim J., Oh A. (2021). Emergent Communication under Varying Sizes and Connectivities. Adv. Neural Inf. Process. Syst..

[62] Tucker M., Li H., Agrawal S., Hughes D., Sycara K.P., Lewis M., Shah J., Beygelzimer A., Dauphin Y., Liang P., Vaughan J.W. (2021). Emergent Discrete Communication in Semantic Spaces. Proceedings of the Advances in Neural Information Processing Systems.

[63] Lake B.M., Salakhutdinov R., Tenenbaum J.B. (2015). Human-level concept learning through probabilistic program induction. Science.

[64] Cao K., Brbić M., Leskovec J. Concept Learners for Few-Shot Learning. Proceedings of the International Conference on Learning Representations (ICLR).

[65] Mao J., Gan C., Kohli P., Tenenbaum J.B., Wu J. (2019). The neuro-symbolic concept learner: Interpreting scenes, words, and sentences from natural supervision. arXiv.

[66] Han C., Mao J., Gan C., Tenenbaum J.B., Wu J. Visual Concept Metaconcept Learning. Proceedings of the Advances in Neural Information Processing Systems (NIPS).

[67] Kim T., Kim S., Bengio Y. (2020). Visual Concept Reasoning Networks. arXiv.

[68] Emruli B., Sandin F., Delsing J. (2015). Vector space architecture for emergent interoperability of systems by learning from demonstration. Biol. Inspired Cogn. Archit..

[69] Wang P.W., Donti P., Wilder B., Kolter Z. Satnet: Bridging deep learning and logical reasoning using a differentiable satisfiability solver. Proceedings of the International Conference on Machine Learning. PMLR.

[70] Feurer M., Klein A., Eggensperger K., Springenberg J.T., Blum M., Hutter F. Efficient and Robust Automated Machine Learning. Proceedings of the NIPS.

[71] Feurer M., Eggensperger K., Falkner S., Lindauer M., Hutter F. (2020). Auto-Sklearn 2.0: The Next Generation. arXiv.

[72] Nichol A., Achiam J., Schulman J. (2018). On first-order meta-learning algorithms. arXiv.

[73] Finn C., Abbeel P., Levine S. Model-agnostic meta-learning for fast adaptation of deep networks. Proceedings of the International Conference on Machine Learning. PMLR.

[74] Wang X., Ye Y., Gupta A. Zero-Shot Recognition via Semantic Embeddings and Knowledge Graphs. Proceedings of the 2018 IEEE/CVF Conference on Computer Vision and Pattern Recognition.

[75] Pourpanah F., Abdar M., Luo Y., Zhou X., Wang R., Lim C., Wang X. (2020). A Review of Generalized Zero-Shot Learning Methods. arXiv.

[76] Shi X., Salewski L., Schiegg M., Akata Z., Welling M. (2020). Relational Generalized Few-Shot Learning. arXiv.

[77] Sun X., Zhang P., Liu J.K., Yu J., Xie W. (2020). Private Machine Learning Classification Based on Fully Homomorphic Encryption. IEEE Trans. Emerg. Top. Comput..

[78] QaisarAhmadAlBadawi A., Chao J., Lin J., Mun C.F., Jie S.J., Tan B.H.M., Nan X., Khin A.M.M., Chandrasekhar V. (2020). Towards the AlexNet Moment for Homomorphic Encryption: HCNN, the First Homomorphic CNN on Encrypted Data with GPUs. IEEE Trans. Emerg. Top. Comput..

